# Robust Fault Detection of Wind Energy Conversion Systems Based on Dynamic Neural Networks

**DOI:** 10.1155/2014/580972

**Published:** 2014-03-11

**Authors:** Nasser Talebi, Mohammad Ali Sadrnia, Ahmad Darabi

**Affiliations:** School of Electrical and Robotic Engineering, University of Shahrood, P.O. Box 3619995161, Shahrood, Iran

## Abstract

Occurrence of faults in wind energy conversion systems (WECSs) is inevitable. In order to detect the occurred faults at the appropriate time, avoid heavy economic losses, ensure safe system operation, prevent damage to adjacent relevant systems, and facilitate timely repair of failed components; a fault detection system (FDS) is required. Recurrent neural networks (RNNs) have gained a noticeable position in FDSs and they have been widely used for modeling of complex dynamical systems. One method for designing an FDS is to prepare a dynamic neural model emulating the normal system behavior. By comparing the outputs of the real system and neural model, incidence of the faults can be identified. In this paper, by utilizing a comprehensive dynamic model which contains both mechanical and electrical components of the WECS, an FDS is suggested using dynamic RNNs. The presented FDS detects faults of the generator's angular velocity sensor, pitch angle sensors, and pitch actuators. Robustness of the FDS is achieved by employing an adaptive threshold. Simulation results show that the proposed scheme is capable to detect the faults shortly and it has very low false and missed alarms rate.

## 1. Introduction

Nowadays the economy and daily life rely on power distribution networks, such that occurrence of a fault in any components of them can affect performance of the overall system. In general, a fault is a phenomenon that changes the behavior of a system in such a way that it is not able to perform its tasks [1]. A feature, which is very important for every structure, is reliability and it can be insured by removing the earlier weaknesses and faults. One way to achieve reliability is implementation of condition monitoring systems and FDSs. Recently, the problem of fault detection for industrial applications, namely, applications where lives are not at risk, has gained extreme importance. In such systems, user satisfaction and economic issues are important. Among these systems, electrical machines [2, 3], power systems, building ventilation systems, and WECSs [4–15] can be mentioned. Repair actions can be performed in a timely manner without the need for an immediate action and this fact is extremely important for off-shore plants where bad conditions (such as storm) can delay any repair operation for several weeks [16, 17]. Although initially FDSs implementation requires investment, producing energy continuously without any interruptions will compensate for initial investment costs. Hence, an FDS for a WECS has advantages such as avoidance of premature breakdown, reduction of maintenance costs, supervision at remote sites, remote diagnosis, improvement of the capacity factor, and support for further development of a WECS [16].

In general, fault detection techniques can be categorized into two categories: hardware redundancy (HR) and analytical redundancy (AR). Furthermore the AR techniques can be classified as quantitative model-based methods and qualitative model-based ones. In recent years, extensive researches have been performed on quantitative model-based methods [18, 19] and qualitative model-based methods [20–22]. Generally these methods can be classified as observer-based, signal processing, expert system, and artificial intelligence approaches. Over the past two decades, artificial neural networks have been studied extremely by researchers and they have been used successfully for modeling and control of dynamical systems [20, 23]. Also they were used to design FDSs [20, 22]. Among the many structures for neural networks, two notable structures are the feedforward and recurrent ones. Feedforward networks are used typically for pattern recognition purposes, while recurrent ones are used to prepare dynamical model of a process. Among neural networks that have been used frequently in FDSs, multilayer feedforward neural networks (MFNNs), radial basis function neural (RBFN) networks, and globally and locally recurrent networks can be mentioned.

In structure of recurrent neural networks, dynamic neurons replace the standard ones. In one type of dynamic neurons, the dynamics are applied by introducing an IIR filter into the neuron structure. This network structure does not have any global feedbacks. In fact, this network has a structure that is somewhere in between a feedforward and a globally recurrent structure [20]. The recurrent neural network with a neuron model using the IIR filter has been used successfully for modeling, fault detection, and time series prediction. In [20, 24] this kind of network was used for fault detection of a sugar evaporation process. Also in [20], applications for fault detection by using the mentioned network were presented. This network was utilized to detect faults in a fluid catalytic cracking and a DC motor. In [2], this network was used for fault detection and isolation of induction motors. In [25], by using this network, an FDS for protecting and monitoring systems of power networks was designed in such a way that reliability of the power grid has risen up. In [26], by combining the RBFN and the neuron model with the IIR filter, a network was proposed and it was used to predict time series. In [27, 28], it was utilized to predict both the wind speed and power in wind farms. Finally, in [29], it was used to detect faults of a turbocharger.

In addition, fault detection of the WECSs has attracted the attention of many researchers in recent years. For instance, in [4, 7], using a linear model of mechanical parts of a wind turbine (electrical parts were neglected), a fault-tolerant controller was presented by utilizing the linear parameter varying control which was replaced with the reference controller. In these two researches, the occurred faults were diagnosed and then the controller was reconfigured. In [5], fault detection of a wind turbine was performed using the linear model of the mechanical parts and only occurrence of a fault was considered. In [6], faults of the induction generator were considered. Also the linear model of the mechanical parts was used to detect two categories of faults using the Kalman-filter in [8]. Utilizing the supervisory control and data acquisition (SCADA) in [9], the occurred faults in a wind turbine were detected. In [10] by using the data-mining approach, bearing faults were detected. In [11], utilizing the real data obtained from a condition monitoring system, faults of the braking system of a wind turbine were detected. In [12], a fault tolerant controller was proposed using fuzzy observers. In [14], utilizing the Set-Membership approach and a model for the mechanical parts, various faults of a wind turbine were detected. Also in [15] numbers of faults were detected using a model for the mechanical parts and the counter-based residual thresholding method.

As it is obvious, in researches that have been performed so far, either a small number of faults have been studied, or the WECS has not been modeled completely, or the linear models have been used to design the FDS. It is obvious that using the more accurate nonlinear model will lead us to the results which are closer to the real ones. Another important issue is robustness of the proposed scheme. Robustness can be achieved either by active approaches or by passive ones [20]. Active approaches consider the desired robustness from the beginning of the design procedure [30–32], while passive approaches utilize adaptive thresholds architecture in decision making block to achieve the desired robustness [20]. In this paper, by utilizing a comprehensive nonlinear model of the WECS, an FDS is suggested which has the capability to detect faults in the generator's angular velocity sensor, pitch sensors, and pitch actuators. The presented FDS is composed of the dynamic neural network with the IIR filter as the dynamic neuron model. Additionally, an adaptive threshold is employed to achieve the anticipated robustness. The proposed FDS can be utilized to detect the faults in other parts of the WECS.

The structure of this paper is organized as follows: dynamic model of the WECS is presented in Section 2. In Section 3, the RNN with the IIR filter as neuron model is introduced.  Section 4 presents the design procedure of the FDS. In Section 5, the robustness of the proposed FDS is investigated.

## 2. Dynamic Model of the WECS

**Figure 1:**
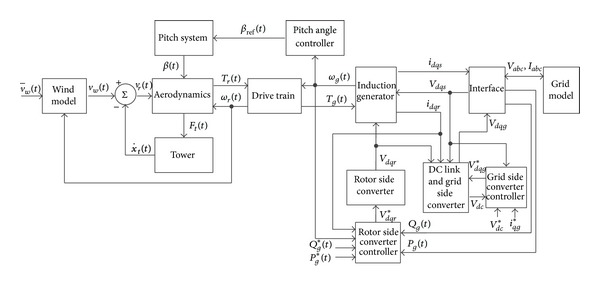
Block Diagram of the dynamic model of the WECS.

The block diagram of the considered model in this paper is shown in Figure 1. In this figure, the yaw mechanism is neglected and wind speed is the exogenous input. The aerodynamic torque of the turbine's rotor, *T*_*r*_(*t*), is transferred to the generator through the drive train. The drive train includes a high speed shaft, a low speed shaft, and a gearbox. The induction generator converts the mechanical energy to the electrical one and is connected to the power grid. An interface is used for calculation of the active and reactive generated power. The grid model includes a local load, transformers, transmission lines, and the infinite-bus at the end. Converters, a dc link, rotor, and grid side controllers are modeled in this study.

Equations for modeling of the mechanical parts of the WECS are as follows [4, 33–36]:

(1) $$\begin{array}{c} v_{w}\left(t\right)=\bar{v_{w}}\left(t\right)+v_{ws}\left(t\right)+v_{ts}\left(t\right)+v_{tu}\left(t\right), \end{array}$$

where in (1), which is related to the modeling of the wind, *v*_*w*_(*t*) is the wind speed including the tower shadow, wind shear, and turbulence components. In this equation, $\bar{v_{w}}(t)$ is the mean wind speed, *v*_*ws*_(*t*) is the wind shear component, *v*_*ts*_(*t*) is the wind speed tower shadow component, and *v*_*tu*_(*t*) is the wind speed turbulence component. Consider

(2) $$\begin{array}{c} P_{r}\left(t\right)=0.5\rho Av_{r}^{3}\left(t\right)C_{p}\left(\lambda \left(t\right),\beta \left(t\right)\right),\\ T_{r}\left(t\right)=\frac{P_{r}\left(t\right)}{\omega_{r}\left(t\right)}=\frac{1}{\omega_{r}\left(t\right)}0.5\rho Av_{r}^{3}\left(t\right)C_{p}\left(\lambda \left(t\right),\beta \left(t\right)\right). \end{array}$$

Equations (2) are used to model wind turbine's aerodynamic, where *P*_*r*_(*t*) is the captured power by the turbine's rotor, *β*(*t*) is the pitch angle, *λ*(*t*) is the tip-speed ratio, *C*_*p*_(*λ*(*t*), *β*(*t*)) is the power coefficient, *A* is the rotor swept area, *v*_*r*_(*t*) is the rotor effective wind speed, *ρ* is the air density, and *T*_*r*_(*t*) is the aerodynamic torque which is applied to the turbine's rotor. Equation (3) represents the aerodynamic thrust where *C*_*t*_(*λ*(*t*), *β*(*t*)) is the thrust coefficient. *C*_*p*_(*λ*(*t*), *β*(*t*)) and *C*_*t*_(*λ*(*t*), *β*(*t*)) can be utilized by look-up tables in simulations. Consider

(3) $$\begin{array}{c} F_{t}\left(t\right)=0.5\rho Av_{r}^{2}\left(t\right)C_{t}\left(\lambda \left(t\right),\beta \left(t\right)\right). \end{array}$$

By drive train, the aerodynamic torque is transferred to the generator. The gearbox scales up the rotational speed to the required generator's angular velocity by a factor that is called gear ratio. Equations (4) are used to model the drive train which includes a low speed shaft, a gearbox, and a high speed shaft. In these equations, *J*_*r*_ is the inertia of the rotor, *T*_*r*_ is the torque acting on the low speed shaft, *ω*_*r*_ is the turbine's rotor speed, *K*_*dt*_ is the spring stiffness coefficient of a massless viscously damped rotational spring, *B*_*dt*_ is viscous damping parameter, *N*_*g*_ is the gear ratio, *J*_*g*_ is the inertia of the gearbox, high speed shaft, and generator, *T*_*g*_ is the generator torque, and *ω*_*g*_ is the rotational speed of the generator's rotor. Consider

(4) $$\begin{array}{c} J_{r}{\dot{\omega }}_{r}=T_{r}-K_{dt}\theta_{\delta }-B_{dt}{\dot{\theta }}_{\delta },\\ J_{g}N_{g}{\dot{\omega }}_{g}=-T_{g}N_{g}+K_{dt}\theta_{\delta }+B_{dt}{\dot{\theta }}_{\delta },\\ {\dot{\theta }}_{\delta }=\omega_{r}-\frac{\omega_{g}}{N_{g}}. \end{array}$$

The thrust makes the tower to sway back and forth. The tower is modeled by a mass-spring-damper according to (5). In this equation, *F*_*th*_(*t*) is the force acting on the tower at hub height, *B*_*t*_ is the tower damping coefficient, *K*_*t*_ is the tower spring coefficient, *M*_*t*_ is the top mass of the tower, and *x*_*t*_(*t*) is the displacement of the nacelle from its equilibrium position. The swaying of the tower changes the effective wind speed seen on the turbine's rotor. The rotor effective wind speed is modeled using (6). Consider the following:

(5) $$\begin{array}{c} M_{t}{\ddot{x}}_{t}\left(t\right)=F_{th}\left(t\right)-B_{t}{\dot{x}}_{t}\left(t\right)-K_{t}x_{t}\left(t\right), \end{array}$$

(6) $$\begin{array}{c} v_{r}\left(t\right)=v_{w}\left(t\right)-{\dot{x}}_{t}\left(t\right). \end{array}$$

The pitch system is a hydraulic system and it can be modeled using (7) where *β*_ref_(*t*) is the reference pitch angle, *ω*_*n*_ is the natural frequency of the pitch actuator model, and *ζ* is the damping ratio of the pitch actuator model. Equation (7) explains the operation of the pitch actuators when it operates within limitations, so physical limitations should be considered in modeling:

(7) $$\begin{array}{c} \ddot{\beta }\left(t\right)=-2\zeta \omega_{n}\dot{\beta }\left(t\right)-\omega_{n}^{2}\beta \left(t\right)+\omega_{n}^{2}\beta_{\text{ref}}\left(t\right). \end{array}$$

Equations for modeling of the electrical parts of the WECS are as follows [37–39]:

(8) $$\begin{array}{c} V_{qs}=R_{s}i_{qs}+\frac{d}{dt}\varphi_{qs}+\omega_{s}\varphi_{ds},\\ V_{ds}=R_{s}i_{ds}+\frac{d}{dt}\varphi_{ds}-\omega_{s}\varphi_{qs},\\ V_{qr}=R_{r}i_{qr}+\frac{d}{dt}\varphi_{qr}+\left(\omega_{s}-\omega_{r}\right)\varphi_{dr},\\ V_{dr}=R_{r}i_{dr}+\frac{d}{dt}\varphi_{dr}-\left(\omega_{s}-\omega_{r}\right)\varphi_{qr}. \end{array}$$

Equations (8) are stator and rotor voltages of the induction machine in the synchronous reference frame. In this model, the synchronous reference frame was used to transform variables from the *abc* frame to the *dq* reference frame. All electrical variables are referred to the stator. In these equations, *R*_*s*_ and *R*_*r*_ are stator and rotor resistance, respectively, *ω*_*s*_ is the synchronous angular velocity, *ω*_*r*_ is the electrical angular velocity, *φ*_*ds*_ and *φ*_*qs*_ are the stator *d* and *q* axis fluxes, and *φ*_*dr*_ and *φ*_*qr*_ are the rotor *d* and *q* axis fluxes. Consider the following:

(9) $$\begin{array}{c} \varphi_{qs}=L_{s}i_{qs}+L_{m}i_{qr},\\ \varphi_{ds}=L_{s}i_{ds}+L_{m}i_{dr},\\ \varphi_{qr}=L_{r}i_{qr}+L_{m}i_{qs},\\ \varphi_{dr}=L_{r}i_{dr}+L_{m}i_{ds}. \end{array}$$

Also the stator and rotor fluxes are expressed by (9), where *L*_*s*_ = *L*_*ls*_ + *L*_*m*_ is the total stator inductance and *L*_*r*_ = *L*_*lr*_ + *L*_*m*_ is the total rotor inductance. The electromagnetic torque is expressed by (10) where *p* is the number of pole pairs. Consider

(10) $$\begin{array}{c} T_{e}=1.5p\left(\varphi_{ds}i_{qs}-\varphi_{qs}i_{ds}\right). \end{array}$$

Two equations of the mechanical parts of the machine are as (11) where *H* is the combined rotor and load inertia constant, *F* is the combined rotor and load viscous friction coefficient, *T*_*g*_ is the mechanical torque, *ω*_*g*_ is the angular velocity of the rotor, and *θ*_*g*_ is the rotor angular position:

(11) $$\begin{array}{c} \frac{d}{dt}\omega_{g}=\frac{1}{2H}\left(T_{e}-F\omega_{g}-T_{g}\right),\\ \frac{d}{dt}\theta_{g}=\omega_{g}. \end{array}$$

Equations (12) can be used to model the grid side converter (GSC) and DC link capacitor, where *R*_*g*_ and *L*_*g*_ are the resistance and inductance of the circuit between the GSC and the grid, *C* is the DC link capacitance, *V*_*dc*_ is the capacitor voltage, *P*_*r*_ is the active power exchanged between the rotor and the rotor side converter (RSC), and *P*_*g*_ is the output active power of the GSC:

(12) $$\begin{array}{c} V_{dg}=R_{g}i_{dg}+L_{g}\frac{d}{dt}i_{dg}-\omega_{s}L_{g}i_{qg}+V_{ds},\\ V_{qg}=R_{g}i_{qg}+L_{g}\frac{d}{dt}i_{qg}+\omega_{s}L_{g}i_{dg}+V_{qs},\\ P_{g}=\frac{3}{2}\left(V_{ds}I_{dg}+V_{qs}I_{qg}\right),\\ \frac{dV_{dc}}{dt}=\frac{P}{V_{dc}C}=\frac{P_{r}-P_{g}}{V_{dc}C}=\frac{P_{e}-P_{s}-P_{g}}{V_{dc}C}. \end{array}$$

The WECS contains three categories of controllers: RSC controller, GSC controller, and pitch angle controller. To control the RSC, the following equation can be used [39]:

(13) $$\begin{array}{c} V_{dr}^{*}=\sigma L_{r}V_{dr}^{\prime }+R_{r}i_{dr}-s\omega_{s}\sigma L_{r}i_{qr}-s\omega_{s}\left(\frac{L_{m}}{L_{s}}\right)\varphi_{qs},\\ V_{qr}^{*}=\sigma L_{r}V_{qr}^{\prime }+R_{r}i_{qr}+s\omega_{s}\sigma L_{r}i_{dr}+s\omega_{s}\left(\frac{L_{m}}{L_{s}}\right)\varphi_{ds}, \end{array}$$

where control voltages *V*_*dr*_′ and *V*_*qr*_′ can be obtained using the PI controllers. These voltages are calculated by comparing *i*_*dr*_ and *i*_*qr*_ currents with reference *i*_dr_* and *i*_qr_* as follows:

(14) $$\begin{array}{c} V_{dr}^{\prime }=\frac{di_{dr}}{dt}=K_{P1}\left(i_{dr}^{*}-i_{dr}\right)+K_{I1}\int \left(i_{dr}^{*}-i_{dr}\right)dt,\\ V_{qr}^{\prime }=\frac{di_{qr}}{dt}=K_{P1}\left(i_{qr}^{*}-i_{qr}\right)+K_{I1}\int \left(i_{qr}^{*}-i_{qr}\right)dt, \end{array}$$

where *K*_*P*1_ is the proportional gain and *K*_*I*1_ is the integral gain of the PI controller. The following equations can be used to control the GSC [39]:

(15) $$\begin{array}{c} V_{dg}^{*}=R_{g}i_{dg}+L_{g}V_{dg}^{\prime }-\omega_{s}L_{g}i_{qg}+V_{ds},\\ V_{qg}^{*}=R_{g}i_{qg}+L_{g}V_{qg}^{\prime }+\omega_{s}L_{g}i_{dg}+V_{qs}, \end{array}$$

where the control voltages *V*_*dg*_′ and *V*_*qg*_′ are obtained using the PI controller as follows:

(16) $$\begin{array}{c} V_{dg}^{\prime }=\frac{di_{dg}}{dt}=K_{P2}\left(i_{dg}^{*}-i_{dg}\right)+K_{I2}\int \left(i_{dg}^{*}-i_{dg}\right)dt,\\ V_{qg}^{\prime }=\frac{di_{qg}}{dt}=K_{P2}\left(i_{qg}^{*}-i_{qg}\right)+K_{I2}\int \left(i_{qg}^{*}-i_{qg}\right)dt. \end{array}$$

The pitch angle controller is responsible for increment or decrement of the pitch angle and it can be implemented by a PI controller. This controller acts on the difference between the reference angular velocity and the measured angular velocity of the generator. In modeling of the WECS, it is usual to neglect the dynamics of the sensors, because they are much faster than the dynamics of the wind turbine. The only expectation is the anemometer which is modeled as a first-order low-pass filter with a half a second time constant [3]. In this study, all measured signals were emulated by adding zero-mean Gaussian distributed noise to deterministic values.

## 3. Dynamic Neural Network

**Figure 2:**
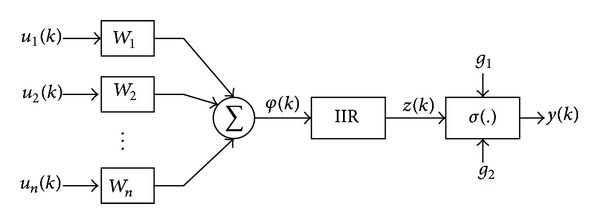
Structure of the neuron model with the IIR filter.

In recurrent neural networks, the dynamic neurons replace the standard static ones. In the structure which is utilized in this paper, dynamics are created by introducing an IIR filter into the neuron architecture where the neuron reproduces its own past inputs and activations by using the input signal *u*_*i*_(*k*), for *i* = 1,2,…, *n* and the output signal *y*(*k*). The structure of the considered neuron model is shown in Figure 2. First the weighted sum of inputs is calculated according to the following equation [20, 29]:

(17) $$\begin{array}{c} \varphi \left(k\right)=\overset{n}{\underset{i=1}{\sum }}w_{i}u_{i}\left(k\right). \end{array}$$

The weights of this network are such as the weights of static feedforward networks. The weights and activation function are responsible for approximation properties of the model. The IIR filters are linear dynamic systems of different orders which consist of feedback and feedforward paths weighted by the weights *a*_*i*_, *i* = 1,2,…, *r* and *b*_*i*_, *i* = 0,1,…, *r*, respectively. The behavior of this linear system can be expressed by the following equation:

(18) $$\begin{array}{c} z\left(k\right)=\overset{r}{\underset{i=0}{\sum }}b_{i}\varphi \left(k-i\right)-\overset{r}{\underset{i=1}{\sum }}a_{i}z\left(k-i\right), \end{array}$$

where *φ*(*k*) is the filter input and *z*(*k*) is the filter output. Eventually the output of the neuron is obtained by the following equation:

(19) $$\begin{array}{c} y\left(k\right)=\sigma \left(g_{2}\left(z\left(k\right)-g_{1}\right)\right), \end{array}$$

where *σ*(·) is a nonlinear activation function and *g*_1_ and *g*_2_ are the bias and the slope parameters of the activation function. There are different methods to train these networks; the three major methods are extended dynamic back propagation (EDBP), adaptive random search (ARS), and simultaneous perturbation stochastic approximation (SPSA). Each of these methods has its own advantages and disadvantages. The ARS method was used in this paper to train the network. The advantage of this method is that it is easy to implement and it has very wide applicability. The information required to implement this method is only the input-output data, where the parameters' vector *θ* is the input and the cost function *J*(*θ*) is the output. All network parameters can be represented by the parameters' vector *θ*. The main purpose of training is to adjust the elements of the vector *θ* so that the cost function is minimized as follows [20]:

(20) $$\begin{array}{c} \theta^{*}=\underset{\theta \in \mathbb{C}}{\min}\;J\left(\theta \right), \end{array}$$

where θ* is the optimal network parameter vector, *J* : ℝ^*p*^ → ℝ^1^ is the cost function to be minimized, *p* is the dimension of the vector *θ*, and *ℂ*⊆ℝ^*p*^ is the constraint set defining the permissible values for the parameters *θ*. The cost function can be defined as follows:

(21) $$\begin{array}{c} J\left(l;\theta \right)=\frac{1}{2}\overset{N}{\underset{k=1}{\sum }}{\left(y_{d}\left(k\right)-y\left(k;\theta \right)\right)}^{2}, \end{array}$$

where *y*_*d*_(*k*) and *y*(*k*; *θ*) are the desired output of the network and the actual response of the network on the given input pattern *u*(*k*), *N* is the dimension of the training set, and *l* is the iteration index. The above cost function should be minimized based on a given set of input-output patterns. In the ARS method, it is not required to compute the gradient of *J*.  Table 1 represents the ARS training algorithm [20]. Assuming that the sequence of solutions ${\hat{\theta }}_{0},{\hat{\theta }}_{1},\ldots ,{\hat{\theta }}_{k}$ is obtained already, to achieve the next point ${\hat{\theta }}_{k+1}$, the following equation is used [20]:

(22) $$\begin{array}{c} {\hat{\theta }}_{k+1}={\hat{\theta }}_{k}+r_{k}, \end{array}$$

where ${\hat{\theta }}_{k}$ is the estimate of θ* at the *k*th iteration and *r*_*k*_ is the perturbation vector generated randomly according to the normal distribution *𝒩*(0, *v*). The new solution ${\hat{\theta }}_{k+1}$ is accepted when the cost function $J({\hat{\theta }}_{k+1})$ is smaller than $J({\hat{\theta }}_{k})$, otherwise ${\hat{\theta }}_{k+1}={\hat{\theta }}_{k}$. In order to start the optimization process, it is necessary to specify the initial value ${\hat{\theta }}_{0}$ and the variance *υ*. Assuming θ* is a global minimum that should be found, when ${\hat{\theta }}_{k}$ is far away from θ*, *r*_*k*_ should have a large variance to allow large displacements. This causes to escape local minima. However, when ${\hat{\theta }}_{k}$ is close to θ*, *r*_*k*_ should have a small variance to allow precise exploration of the parameter space. Far from its convenience of training, the algorithm has the global convergence property and adaptive parameters of the algorithm reduce the possibility of trapping in local minima [20].

## 4. FDS Scheme

In this research, among the possible faults of the WECS, the pitch sensors, generator's angular velocity sensor, and pitch actuators faults were studied; because according to the information of the fault analysis which is given in [4], these three categories of faults have more severity and occurrence indices than the other faults. Internal faults of the generator and converter were not studied in this research in order to prevent the research from becoming too complex and extensive. It is also assumed that no fault has occurred in the control systems of the WECS and they continue to function properly. Thus it can be said that the normal operation conditions of the WECS are as follows:

- the control systems are healthy;
- any type of faults has not occurred in the WECS;
- any fault has not occurred in the power grid connected to the WECS.

Also the faulty operation conditions are as follows:

- the control systems are healthy and they continue to act normally when a fault has occurred;
- one of the faults in the pitch sensors, pitch actuators, or generator's angular velocity sensor has occurred; it is assumed that these faults do not occur at the same time;
- any fault has not occurred in the power grid connected to the WECS.

In design of the FDS, studying the two signals of the generator's angular velocity and pitch angles causes to detect the faults in the pitch system and generator's angular velocity sensor. According to the model of the WECS, both mentioned signals can be considered as a nonlinear function of the turbine's rotor speed and the measured wind speed. These two measurements are available from the healthy sensors. Because the closed-loop control system performance varies under different wind speeds, the wind speed is regarded as an input of the nonlinear functions. So the following relations can be considered:

(23) $$\begin{array}{c} \omega_{g}=h_{1}\left(V_{w},\omega_{r}\right), \end{array}$$

(24) $$\begin{array}{c} \beta_{1,2,3}=h_{2}\left(V_{w},\omega_{r}\right), \end{array}$$

where *h*_1_(·) and *h*_2_(·) are nonlinear functions. Thus according to the measured values of the signals in the input of these functions, their outputs can be estimated by the mentioned dynamic neural network. These neural models can be used to emulate the normal system behavior. The neural models are then placed in parallel with the system and fault detection is acquired by comparing the outputs of the neural models with the real system outputs. First, the generator's angular velocity is modeled by neural network. The process to be modeled is described by (23). This process has two inputs and one output. The training process was carried out off-line using the ARS algorithm. The learning set consisted of 1000 samples for different mean wind speeds whilst the testing set consisted of 2000 samples for the mean wind speed of 16 m/s. First these samples were converted to the p.u. according to their base values and then the p.u. values were used to train the network. The best model was selected by using two information criteria. These criteria are the Akaike information criterion (AIC) and the final prediction error (FPE). The AIC determines the model complexity by minimizing an information theoretical function, *f*_AIC_, which is defined as follows [20]:

(25) $$\begin{array}{c} f_{\text{AIC}}=\log\left(J\right)+\frac{2K}{N}, \end{array}$$

where *J* is the sum of squared errors between the desired output, *y*_*i*_^*d*^, and the network output *y*_*i*_, *N* is the number of samples used in the computation of *J*, and *K* is the number of model parameters. Another criterion is the FPE, which selects the model order minimizing the function FPE, *f*_FPE_, which is defined as follows [20]:

(26) $$\begin{array}{c} f_{\text{FPE}}=\frac{J\left(1+\left(K/N\right)\right)}{\left(1-\left(K/N\right)\right)}. \end{array}$$

**Figure 3:**
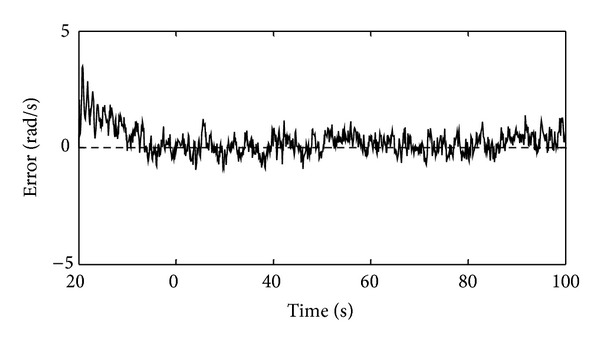
Residual signal of the generator's angular velocity sensor resulting from the WECS simulation for the mean wind speed of 14 m/s in normal conditions.

The results of the appropriate network structure selection to model *ω*_*g*_ are presented in Table 2. In this table *K* is the number of network parameters, *J* is the sum of squared errors, and *N*_*n*,*v*,*s*_^*m*^(*r*) represents the *m* layered dynamic neural network with *n* inputs, *v* hidden neurons, and *s* outputs in which neurons are of the *r* order of the IIR filters. The best results (marked with the bold text) were obtained for the *N*_2,3,1_^2^(2) structure for the training set. However, for the testing set, *N*_2,3,1_^2^(1) structure shows better performance. Eventually the *N*_2,3,1_^2^(1) architecture, which corresponds to the minimum criteria in testing, was selected as optimal to model behaviors of *ω*_*g*_, because this structure has better generalization capability than the one selected for the training set. Each neuron of the dynamic network model has the hyperbolic tangent activation function and is of the first order of the IIR filter.  Figure 3 shows the comparison of the real *ω*_*g*_ and the estimated *ω*_*g*_ by the designed dynamic neural network in the normal operation conditions. As it is obvious, the designed neural network estimates *ω*_*g*_ desirably and estimation error is minimal. All simulations were carried out in Matlab/Simulink version R2008a environment. The parameters of the system are presented in the appendix.

**Figure 4:**
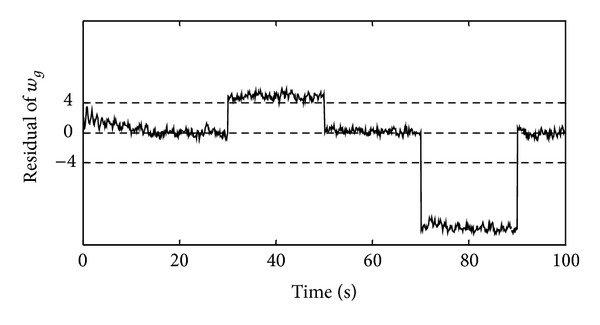
The residual of the generator's angular velocity for the mean wind speed of 14 m/s during occurrence of the abrupt proportional fault in the generator's angular velocity sensor.

In order to form the FDS of the generator's angular velocity, two types of faults were considered which will be discussed below. In the basic FDS, the constant threshold was used to evaluate the residual signals. This threshold level was considered equal to ±4 rad/s. In the first simulation, the +2% abrupt proportional fault was introduced at time instant 30 s for duration of 20 s and the −5% abrupt proportional fault was introduced at time instant 70 s for duration of 20 s.  Figure 4 shows the simulation result in this case.

According to Figure 4, in the case of the generator's angular velocity deviation from its healthy value, a residual is obtained by neural model estimator which is compared with the threshold level; hence the fault occurrence is detectable. In this figure, between the moments of 30 to 50 seconds and 70 to 90 seconds, the obtained residual passes the ±4 rad/s threshold level and this event indicates that a fault has occurred in the generator's angular velocity sensor. As a result, it can be argued that the FDS has the capability of detecting the abrupt proportional faults in the generator's angular velocity sensor.

**Figure 5:**
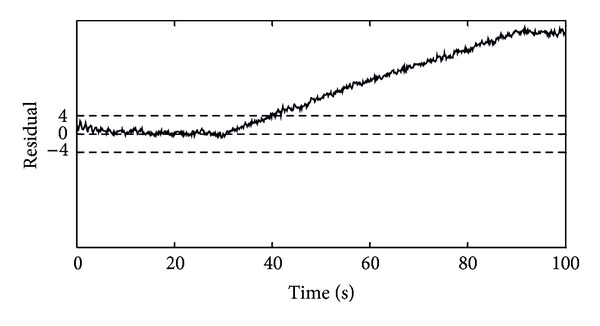
The residual of generator's angular velocity for the mean wind speed of 12 m/s during occurrence of the incipient proportional fault in the generator's angular velocity sensor.

The second simulation was performed when an incipient proportional fault occurred in the generator's angular velocity sensor. In this case, output of the generator's angular velocity sensor was deviated from its real value at time instant 30 s until it reached to 1.1 times greater than its real value at time instant 90 s; that is, during 60 seconds, an incipient fault was introduced in the generator's angular velocity sensor. In this case, the simulation result is presented in Figure 5. According to Figure 5, the FDS has the ability to detect the introduced incipient fault at time instant 40.7 s. It means that it takes about 11 seconds until the FDS detects this type of fault which is so desirable in detection of incipient faults. According to the obtained results for the abrupt proportional fault, incipient proportional fault, and fixed output fault (which is a special case of the incipient fault), it can be concluded that the designed FDS can detect faults well in different wind speeds (which affects the dynamics of the system). This FDS has capability of the early detection of faults. The significant point in this system is a very low number of false alarms which is desired in any FDSs.

**Figure 6:**
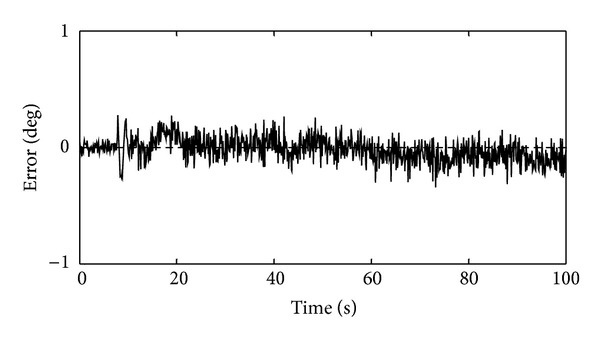
Residual signal of the pitch system of the blade 1 resulting from the WECS simulation for the mean wind speed of 14 m/s in normal conditions.

The next phase in design of the FDS is related to the pitch system fault detection. In the pitch system, the pitch sensors and actuators are subjected to the faults. Therefore, the pitch system should be modeled by neural network. The process to be modeled is described by (24). The training process was carried out off-line using the ARS algorithm. The learning set consisted of 1000 samples for different mean wind speeds and the testing set consisted of 2000 samples for the mean wind speed of 16 m/s. First these samples were converted to the p.u. values according to their base values and then the p.u. values were used to train the network. The best model was selected by using the AIC and FPE criteria. The results of the appropriate network structure selection are presented in Table 3. The best results were obtained for the *N*_2,3,1_^2^(2) structure for the both training and testing sets. Thus, the *N*_2,3,1_^2^(2) architecture was selected as optimal to model behaviors of the pitch system in the normal operation conditions. Each neuron of the dynamic network model has the hyperbolic tangent activation function and is of the second order of the IIR filter.  Figure 6 shows the comparison of the real *β*_1_ and the estimated *β*_1_ by the designed dynamic neural network in normal operation conditions. As it can be seen, the estimation error is minimal and the designed neural network has good ability to mimic the pitch angles behaviors.

**Figure 7:**
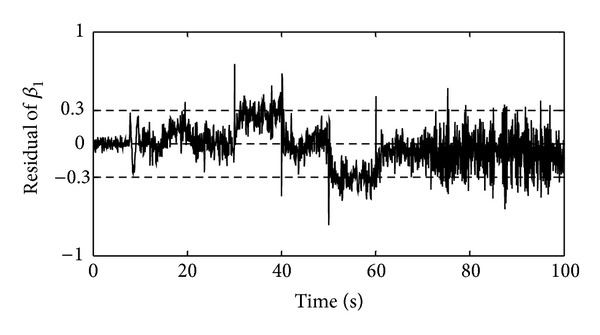
Residual evaluation by the constant threshold for the mean wind speed of 14 m/s during occurrence of three categories of faults in the pitch system.

In order to form the FDS of the pitch system, three types of faults were considered. Faults of the pitch system were categorized such that an increase in the output of the blade 1 pitch sensor (positive bias) was considered as the category 1, a decrease in the output of the blade 1 pitch sensor (negative bias) was considered as the category 2, and faults of the pitch actuator which were modeled by changing the *ζ* and *ω*_*n*_ were considered as the category 3. To study the residual signal, the +0.75° bias fault was introduced at time instant 30 s for duration of 10 s, the −1° bias fault was introduced at time instant 50 s for duration of 10 s, and the pitch actuator fault which is an abrupt fault was introduced at time instant 70 s for duration of 30 s.  Figure 7 shows the residual signal under these conditions. In the basic FDS, the constant threshold can be used to evaluate the residual signal. This threshold level was considered equal to ±0.3°. As it can be seen, the occurred faults can be detected using the constant threshold. According to Figure 7, it takes 1.3 s, 2.1 s, and 2.5 s to detect the positive bias, the negative bias, and the pitch actuator faults, respectively. Using this residual and the constant threshold, the minimum detectable fault is equal to ±0.5°. Obviously, the threshold value can be reduced to further speed up the detection process; however, by reducing the detection time, false alarms will increase. In fact, a tradeoff should be considered between the detection time and false alarms rate.

**Figure 8:**
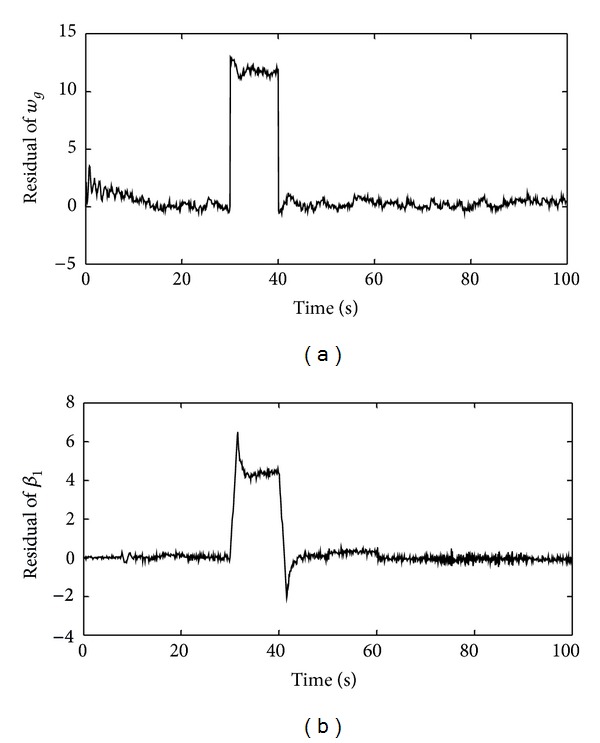
The residual signals for occurrence of three types of faults: (a) the residual signal of the generator's angular velocity sensor and (b) the residual signal of the pitch system of the blade 1.

To evaluate the FDS performance in the presence of the pitch sensors and actuators and the generator's angular velocity sensor faults, first it is necessary to point out that occurrence of a fault in the angular velocity sensor of the generator affects the residual signal of the pitch system. To investigate this issue, the +5% proportional fault in the generator's angular velocity sensor was introduced at time instant 30 s for duration of 10 s, the +1° bias fault in the blade 1 pitch sensor was introduced at time instant 50 s for duration of 10 s, and the pitch actuator fault was introduced at time instant 70 s for duration of 20 s.  Figure 8 represents the residual signals which were obtained from the FDS. As it can be seen from the results, by introducing a fault in the generator's angular velocity sensor, the residual signal of the blade 1 pitch sensor was affected and changed. Conversely, by introducing a fault in the pitch system, the residual signal of the generator's angular velocity was not affected.

Consequently, to form an integrated FDS, it should be considered that if both of the residuals have passed the threshold level, it means that a fault has occurred in the generator's angular velocity sensor. But if only the residual of the blade 1 pitch angle has passed the threshold level, it means that a fault has occurred in the pitch system. The general algorithm for detection of the studied faults in the WECS is suggested as follows:

1. if both of the residuals of the generator's angular velocity sensor and pitch system remain within the threshold level, no fault has occurred in the WECS;
2. if both of the residuals of the generator's angular velocity sensor and pitch system pass the threshold level, a fault has occurred in the generator's angular velocity sensor;
3. If the residual of the generator's angular velocity remains within the threshold level but the residual of the pitch system passes the threshold level, a fault has occurred in the pitch system.

**Figure 9:**
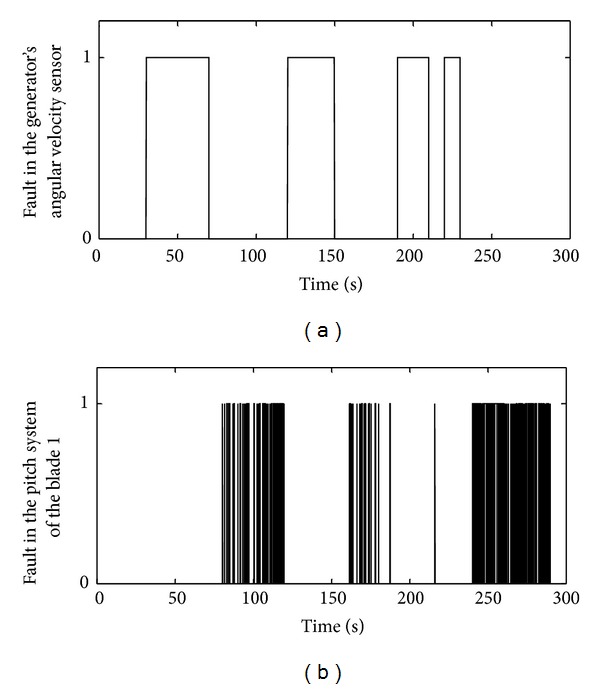
The residual signals under occurrence of the faults specified in Table 4: (a) the residual signal of the generator's angular velocity sensor and (b) the residual signal of the pitch system of the blade 1.

In order to evaluate performance of the FDS together with the proposed algorithm, various faults were introduced to the WECS for duration of 300 s according to Table 4. In this case, the residual signals are shown in Figure 9. In this figure, a value of 1 means occurrence of a fault and a value of zero means that the corresponding element functions properly. As it can be seen from the results, performance of the designed FDS is very good together with the minimal false fault detections. In this figure, there are very few false alarms which are significant and desirable. To reduce the number of false detections, the threshold level can be increased, in order to avoid false alarms due to noises, disturbances, and uncertainties. However an increase in the threshold level will be followed by the sensitivity reduction of the FDS. Always a compromise should be considered to reduce the number of false alarms and to achieve the desired sensitivity when a constant threshold is utilized.

## 5. The Robustness Issue

Since there are disturbances, uncertainties, and measurement noises in practical systems, decision making can be sensitive to them. The mentioned factors cause the robustness issue to be so important in FDSs. The robustness problem in FDSs is to minimize the number of false alarms and to maximize its sensitivity simultaneously. As it is described in [20], robustness of FDSs can be acquired by two general methods:

1. active methods in which the robustness issue is considered from the beginning of the design procedure of the FDS so that it is not sensitive to disturbances, uncertainties, and measurement noises. Generally, these approaches utilize unknown input observers, robust parity equations, and linear parameter varying observers [30–32];
2. passive methods in which the robustness issue is achieved in the decision making component of the FDS. In these approaches, robustness is obtained by an adaptive threshold. As it is stated in [20], the passive method has an advantage over the active one, since the desired robustness can be acquired in spite of uncertain parameters of the model.

To avoid false alarms in passive approaches, the value of the constant thresholds should be selected large enough because of unmodeled dynamics, disturbances, uncertainties, and measurement noises. On the other hand, a large value for the constant threshold causes the sensitivity degradation of the FDS. As it is mentioned earlier, when a constant threshold is utilized, a compromise should be considered to reduce the number of false alarms and to attain the desired sensitivity. Therefore, it is suggested to use the adaptive threshold. In the adaptive threshold, the value of the threshold varies in time according to the obtained information from the residual signal. In this paper, to achieve the robustness of the FDS, the proposed adaptive threshold in [20] is utilized. This adaptive threshold is described by the following equations:

(27) $$\begin{array}{c} T\left(k\right)=t_{\gamma }\overset{-}{\nu }\left(k\right)\pm \overset{-}{m}\left(k\right),\\ \overset{-}{\nu }\left(k\right)=\eta \nu \left(k\right)+\left(1-\eta \right)\nu \left(k-1\right),\\ \overset{-}{m}\left(k\right)=\eta m\left(k\right)+\left(1-\eta \right)m\left(k-1\right), \end{array}$$

where *ν*(*k*) and *m*(*k*) are the variance and mean value of the residual signal for the past *n* samples, respectively, and *η* is the momentum parameter which is considered close to 1. Furthermore, the significance level, *γ*, relates to probability of exceeding the residual signal from a random value, *t*_*γ*_, with *𝒩*(0,1). The following equation describes the significance level [20]:

(28) $$\begin{array}{c} \gamma =P\left(\left|\frac{r-m}{\nu }\right|>t_{\gamma }\right). \end{array}$$

**Figure 10:**
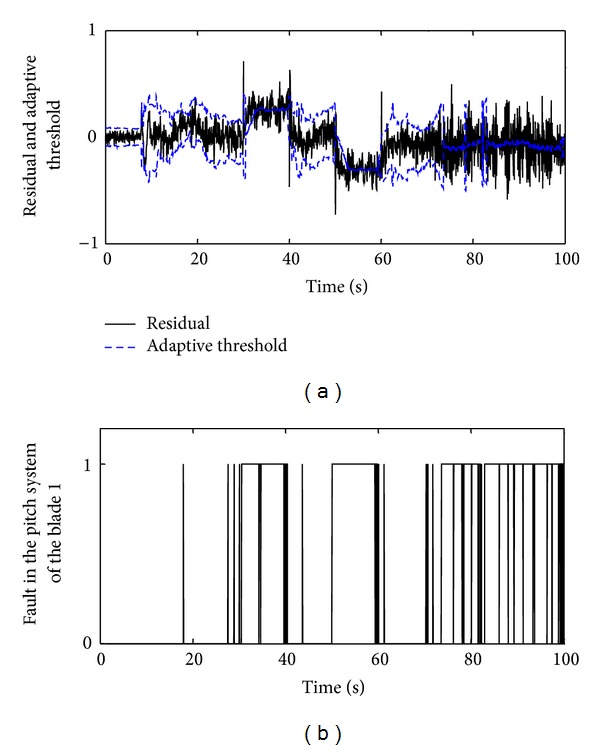
Residual evaluation by the adaptive threshold during occurrence of three categories of faults in the pitch system: (a) the residual and adaptive threshold and (b) decision making by means of the adaptive threshold.

To investigate performance of the defined adaptive threshold, the residual signal in Figure 7 was considered again and the adaptive threshold was calculated. In this case, the simulation result is shown in Figure 10. As it is obvious from the obtained result, the adaptive threshold indicates the satisfactory behaviors. Additionally, the number of false and missed alarms for both the adaptive and constant thresholds is calculated and results are gathered in Table 5. The statistical analysis confirms that the performance of the adaptive threshold (Figure 10) is considerably better than the constant threshold (Figure 7). By using the adaptive threshold, the number of both false and missed alarms is reduced significantly.

Furthermore, to study the efficiency of the presented FDS, the integrated test, which is presented in Table 4, is performed again. To compare the performance of the FDS together with the adaptive threshold and the FDS together with the constant threshold, three indices are utilized. These indices are the number of false and missed alarms and detection time, *t*_detect_.  Table 6 shows the obtained results in this case. It is noteworthy that in Table 6, the number of false alarms is calculated for whole residual signal; however, the number of missed alarm is calculated for each type of fault separately. Simulation results verify the validity of the proposed FDS. The presented FDS together with the adaptive threshold has good sensitivity and the number of its missed alarms is appropriate. Likewise its detection time is very low and favorable.

## 6. Conclusion

In this paper, an FDS was proposed for the WECS. The dynamic model of the WECS was a comprehensive one which contained both the electrical and mechanical parts. Detection of faults was performed in such a way that two neural models were used to emulate the normal system behaviors. They were then placed in parallel with the system and fault detection was acquired by comparing their outputs. The designed FDS can detect faults in the generator's angular velocity sensor, the pitch sensors, and actuators. The FDS employed the dynamic RNN and the ARS method was used to train the network. This kind of dynamic neural networks presented very high ability to estimate both the generator's angular velocity and pitch angle. In order to attain the robustness, it was suggested to use an adaptive threshold because the constant threshold was so sensitive to measurement noises and disturbances. The simulation results for different operation conditions verify that the proposed FDS operates fast, precisely, and accurately and detects the faults appropriately and its false and missed alarms are very low.

## Figures and Tables

**Table 1:** The ARS training algorithm.

Step	Operation
(1) Initialization phase:	${\hat{\theta }}_{0}$, *n*_max⁡_, *υ*_0_ and *J*_min⁡_ should be selected and *n* = 1, ${\hat{\theta }}_{\text{best}}={\hat{\theta }}_{0}$;

(2) Variance selection phase:	*i* = 1, *k* = 1, ${\hat{\theta }}_{k}={\hat{\theta }}_{0}$; while (*i* < 5) while (*k* ≤ 100/*i*) The trial point algorithm is executed; *k* = *k* + 1; end *i* = *i* + 1, *k* = 1, ${\hat{\theta }}_{k}={\hat{\theta }}_{0}$; end

(3) Variance exploration phase:	*k* = 1, ${\hat{\theta }}_{k}={\hat{\theta }}_{\text{best}}$, *i* = *i*_best_; while (*k* ≤ 100) The trial point algorithm is executed; *k* = *k* + 1; end if ((*n* = *n*_max⁡_) or ($J({\hat{\theta }}_{\text{best}})<J_{\min}$)) Break; else ${\hat{\theta }}_{0}={\hat{\theta }}_{\text{best}}$, *n* = *n* + 1 and go to *Step 2*.; end

The trial point algorithm:	*υ*_*i*_ = 10^−*i*^ *υ*_0_, ${{\hat{\theta }}_{k}}^{\prime }={\hat{\theta }}_{k}+r_{k}$; if ($J({{\hat{\theta }}_{k}}^{\prime })\leq J({\hat{\theta }}_{k})$) ${\hat{\theta }}_{k+1}={{\hat{\theta }}_{k}}^{\prime }$; else ${\hat{\theta }}_{k+1}={\hat{\theta }}_{k}$; end if ($J({{\hat{\theta }}_{k}}^{\prime })\leq J({\hat{\theta }}_{\text{best}})$) ${\hat{\theta }}_{\text{best}}={{\hat{\theta }}_{k}}^{\prime }$; *i*_best_ = *i*; end

**Table 2:** The training and testing results for the network structure selection to model *ω*_*g*_.

Network structure	*K*	Training			Testing		
		*J*	*f*_FPE_	*f*_AIC_	*J*	*f*_FPE_	*f*_AIC_
N_2,3,1_^2^(1)	25	0.0282	0.0296	−3.5148	**0.0126**	**0.0129**	**−4.3491**
N_2,4,1_^2^(1)	33	0.0379	0.0405	−3.2068	0.0200	0.0207	−3.8790
N_2,5,1_^2^(1)	41	0.0258	0.0286	−3.5554	0.0151	0.0157	−4.1521
N_2,6,1_^2^(1)	49	0.0521	0.0575	−2.8566	0.0573	0.0602	−2.8105
N_2,7,1_^2^(1)	57	0.0406	0.0455	−3.0900	0.0664	0.0703	−2.6551
N_2,3,1_^2^(1)	31	**0.0268**	**0.0285**	**−3.5574**	0.0134	0.0138	−4.2815
N_2,4,1_^2^(1)	41	0.0417	0.0453	−3.0953	0.0424	0.0442	−3.1196
N_2,5,1_^2^(1)	51	0.0378	0.0419	−3.1734	0.0409	0.0430	−3.1456
N_2,6,1_^2^(1)	61	0.0349	0.0394	−3.2333	0.0327	0.0348	−3.3594
N_2,7,1_^2^(1)	71	0.0795	0.0917	−2.3900	0.0850	0.0913	−2.3941
N_2,3,2,1_^3^(2-2)	50	0.1286	0.1421	−1.9510	0.0365	0.0384	−3.2604
N_2,4,2,1_^3^(2-2)	61	0.0902	0.1019	−2.2837	0.0767	0.0815	−2.5069
N_2,4,3,1_^3^(2-2)	73	0.1135	0.1314	−2.0300	0.0141	0.0152	−4.1886

**Table 3:** The training and testing results for the network structure selection to model *β*_1_.

Network structure	*K*	Training			Testing		
		*J*	*f*_FPE_	*f*_AIC_	*J*	*f*_FPE_	*f*_AIC_
N_2,3,1_^2^(1)	25	0.2447	0.2583	−1.3536	0.2510	0.2574	−1.3573
N_2,4,1_^2^(1)	33	0.1023	0.1099	−2.2083	0.1471	0.1520	−1.8836
N_2,5,1_^2^(1)	41	0.0394	0.0431	−3.1451	0.0821	0.0855	−2.4588
N_2,6,1_^2^(1)	49	0.0422	0.0465	−3.0673	0.0964	0.1012	−2.2902
N_2,7,1_^2^(1)	57	0.0509	0.0561	−2.8799	0.1150	0.1208	−2.1138
N_2,3,1_^2^(1)	31	**0.0304**	**0.0325**	**−3.4261**	**0.0793**	**0.0818**	**−2.5035**
N_2,4,1_^2^(1)	41	0.0781	0.0854	−2.4609	0.1078	0.1123	−2.1865
N_2,5,1_^2^(1)	51	0.0471	0.0526	−2.9450	0.1037	0.1091	−2.2153
N_2,6,1_^2^(1)	61	0.0348	0.0384	−3.2601	0.0873	0.0917	−2.3894
N_2,7,1_^2^(1)	71	0.0457	0.0527	−2.9437	0.0782	0.0936	−2.3686
N_2,3,2,1_^3^(2-2)	50	0.0641	0.0714	−2.6390	0.0952	0.1001	−2.3018
N_2,4,2,1_^3^(2-2)	61	0.0373	0.0426	−3.1566	0.0849	0.0902	−2.4053
N_2,4,3,1_^3^(2-2)	73	0.0282	0.0325	−3.4103	0.1012	0.1089	−2.2177

**Table 4:** Faults specifications in the integrated test.

Location	Type	Start time	Stop time
Generator's angular velocity sensor	Proportional +2% (*f*_1_)	30	70
Pitch actuator of the blade 1	Change in *ζ* and *ω*_*n*_ (*f*_2_)	80	120
Generator's angular velocity sensor	Proportional −10% (*f*_3_)	120	150
Pitch sensor of the blade 1	Positive bias +1° (*f*_4_)	160	180
Generator's angular velocity sensor	Proportional +5% (*f*_5_)	190	210
Generator's angular velocity sensor	Proportional −2% (*f*_6_)	220	230
Pitch sensor of the blade 1	Negative bias −0.8° (*f*_7_)	240	260
Pitch actuator of the blade 1	Change in *ζ* and *ω*_*n*_ (*f*_8_)	265	290

**Table 5:** The number of false and missed alarms.

Threshold type	Number of false alarms	Number of missed alarms
Constant	8	382
Adaptive	5	57

**Table 6:** Evaluation of the suggested FDS for the constant and adaptive thresholds.

Fault type	Number of false alarms		Number of missed alarms		*t*_detect_	
	Constant	Adaptive	Constant	Adaptive	Constant	Adaptive
*f*_ 1_	29: for all of the samples during 300 seconds	13: for all of the samples during 300 seconds	3	2	0.2	0.05
*f*_ 2_			348	74	0.3	0.4
*f*_ 3_			3	3	0.25	0.1
*f*_ 4_			235	41	1.5	0.6
*f*_ 5_			3	1	0.2	0.2
*f*_ 6_			3	2	0.1	0.1
*f*_ 7_			79	11	0.1	0.3
*f*_ 8_			245	39	0.5	0.35

## Appendix

### Parameters of the System

*P* = 2 MW, *V*_*n*_ = 690 v, *f*_*n*_ = 60 Hz, *ω*_*g*,nom⁡_ = 195.8 rad/s, Number of blades = 3, *N*_*g*_ = 85, *K*_*dt*_ = 1.0383 × 10^8^, *B*_*dt*_ = 1.0383 × 10^6^, *h* = 60 m, *R* = 30.56 m, *J*_*r*_ = 8.7 × 10^6^ kg m^2^, *J*_*g*_ = 150 kg m^2^, *M*_*t*_ = 250 × 10^3^ kg, *K*_*t*_ = 5.55 × 10^6^ Nm, *B*_*t*_ = 2.98 × 10^3^ N/m/s, *v*_*w*,cut-in_ = 4 m/s, *v*_*w*,cut-off_ = 25 m/s, *ρ* = 1.225 kg/m^3^, Number of poles = 4, *R*_*s*_ = 0.0069314 p.u., *R*_*r*_ = 0.00906 p.u., *L*_*ls*_ = 0.08083 p.u., *L*_*lr*_ = 0.09934 p.u., *L*_*m*_ = 3.29 p.u., *V*_*dc*,nom⁡_ = 1200 v, *C* = 10000 × 10^−6^ F, *R*_*g*_ = 0.0015 p.u., *L*_*g*_ = 0.15 p.u.
